# Dual-Setting Bone Cement Based On Magnesium Phosphate Modified with Glycol Methacrylate Designed for Biomedical Applications

**DOI:** 10.1021/acsami.3c14491

**Published:** 2023-11-21

**Authors:** Marcin Wekwejt, Maryia Khamenka, Anna Ronowska, Uwe Gbureck

**Affiliations:** † Biomaterials Technology Department, Faculty of Mechanical Engineering and Ship Technology, Gdańsk University of Technology, G. Narutowicza 11/12 Street, 80-233 Gdańsk, Poland; ‡ Scientific Club “Materials in Medicine”, Advanced Materials Centre, Gdańsk University of Technology, G. Narutowicza 11/12 Street, 80-233 Gdańsk, Poland; § Chair of Clinical Biochemistry, Department of Laboratory Medicine, Medical University of Gdańsk, 2x, M. Skłodowskiej-Curie 3a Street, 80-210 Gdańsk, Poland; ∥ Department for Functional Materials in Medicine and Dentistry, University of Würzburg, Pleicherwall 2 Street, D-97070 Würzburg, Germany

**Keywords:** magnesium phosphate, bone cement, 2-hydroxyethyl methacrylate, dual-setting cement, mechanical properties

## Abstract

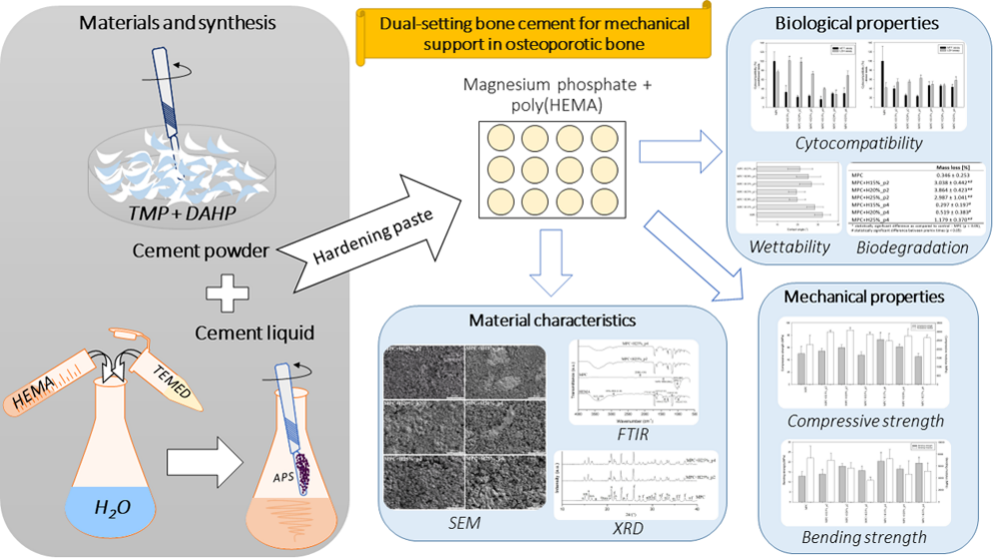

Magnesium phosphate cement (MPC) is a suitable alternative for the currently used calcium phosphates, owing to beneficial properties like favorable resorption rate, fast hardening, and higher compressive strength. However, due to insufficient mechanical properties and high brittleness, further improvement is still expected. In this paper, we reported the preparation of a novel type of dual-setting cement based on MPC with poly(2-hydroxyethyl methacrylate) (pHEMA). The aim of our study was to evaluate the effect of HEMA addition, especially its concentration and premix time, on the selected properties of the composite. Several beneficial effects were found: better formability, shortened setting time, and improvement of mechanical strengths. The developed cements were hardening in ∼16–21 min, consisted of well-crystallized phases and polymerized HEMA, had porosity between ∼2–11%, degraded slowly by ∼0.1–4%/18 days, their wettability was ∼20–30°, they showed compressive and bending strength between ∼45–73 and 13–20 MPa, respectively, and, finally, their Young’s Modulus was close to ∼2.5–3.0 GPa. The results showed that the optimal cement composition is MPC+15%HEMA and 4 min of polymer premixing time. Overall, our research suggested that this developed cement may be used in various biomedical applications.

## 1 Introduction

Bone tissue has an innate regenerative potential; however, there is a limited ability for self-healing of defects caused by complicated injuries, tumor resection, infections, or avascular bone necrosis.^1^ In consequence, there may be a need to perform surgery with the additional use of bone grafts to induce bone regeneration.^2^ Biomaterials are increasingly being used to support the treatment of nonunion of bone or as mechanical reinforcement in osteoporosis.^3^ The ideal bone substitute should be osteoconductive, osteoinductive, and osteogenic as well as ensure adequate mechanical stability of the bone defect.^4^ Moreover, materials are also expected to be injectable, which allows them to be used in minimally invasive surgical procedures.^5^ This feature is met by a specific group of biomaterials called bone cements. These materials typically consist of a powder and a liquid, which form a self-setting paste after mixing.^6^ Two basic groups of cements exist, either based on minerals showing a hydraulic setting reaction or polymeric cements on the basis of poly(methyl methacrylate)—PMMA. PMMA-based cements are bioinert and do not result in bone regeneration. In contrast, calcium phosphate cements are characterized by high bioactivity by adsorption and release of ions contributing to bone regeneration.^7−9^ An alternative to the currently used calcium phosphate cements is magnesium phosphate cement (MPC), which is still in the experimental phase in terms of clinical aspects. Magnesium, due to its unique biological properties, except for use as an implant, was applied as a bioactive coating for scaffolds,^10,11^ bioink for 3D printing,^12^ or active drug delivery system.^13^ Here, in the case of cement, the main advantages of MPC include fast hardening, favorable resorption rate, high initial mechanical strength, appropriate cytocompatibility, and resorption profile *in vivo*, with the potential to promote bone regeneration. They also may have antimicrobial properties for specific cement formulations.^14−16^ The conducted research showed high biocompatibility for MPC, and *in vivo* studies confirmed complete biodegradation after ∼6 months.^17,18^ However, like any biomaterial, it also has some imperfections that should be improved, such as high setting temperature, inadequate washout resistance during application, and limited injectability, and like other mineral cement, MPC is inherently brittle.^19,20^ The solution to overcome those problems is either fiber reinforcement or even more effective modification with an in situ-formed hydrogel phase (“dual setting”) to obtain cements with a pseudoductile fracture behavior, where the stress–strain curve looks similar to those of ductile metals. The above-mentioned strategies were previously tested mainly for calcium phosphate and PMMA cements.^21^ For example, Rödel et al. combined brushite cement with methacrylated poly(ethylene glycol),^22^ Schamel et al. received hybrid cement based on brushite and silk fibroin,^23^ and also Rödel et al. developed brushite-gelatin cement.^24^ Moreover, apatite cements were used as dual-setting systems in combination with methacrylated dextran,^25^ ammonium polyacrylate,^26^ and isocyanate-modified prepolymer.^27^ There is also research on the development of new composite bone cements based on PMMA matrix modified with, i.e., CaP,^28^ carbon nanotubes,^29^ amine-functionalized graphene,^30^ or borosilicate glass.^31^ Further, Rad et al. developed bioactive cement based on three components: PMMA, elastin, and nanohydroxyapatite.^32^ Furthermore, there are also works on α-tricalcium phosphate cement combined with 2-hydroxyethyl methacrylate as representative of the dual-setting group.^33,34^ In all scientific results from the articles mentioned above, attention was paid to the beneficial synergistic effect of ceramic-polymer cement hybrids. There is not much data about dual-setting systems with magnesium phosphate cement, and there are only works available for the modification with water-soluble polymers, such as cross-linked poly(vinyl alcohol),^35^ carboxymethyl chitosan-alginate,^36^ chitosan,^37^ or oxidized-carboxymethyl chitosan.^38^ Dual-setting MPC with simultaneous formation of a hydrogel network from monomers or prepolymers and a cement matrix by a hydraulic reaction is, to the best knowledge of the authors, not yet described. The current work aims to close this gap by developing such a novel dual-setting cement based on 2-hydroxyethyl methacrylate as a monomer and a struvite-forming cement powder. The main goal of our study was to develop a new composite material that could be used in various biomedical applications. Moreover, we will invent the manufacturing technology that will allow us to obtain an optimized bone cement composition with improved functional and mechanical properties. Finally, our development may, in the future, minimize surgically invasive procedures for bone defect treatment.

## 2 Materials and Methods

### 2.1 Cement Preparation

**Figure 1:**
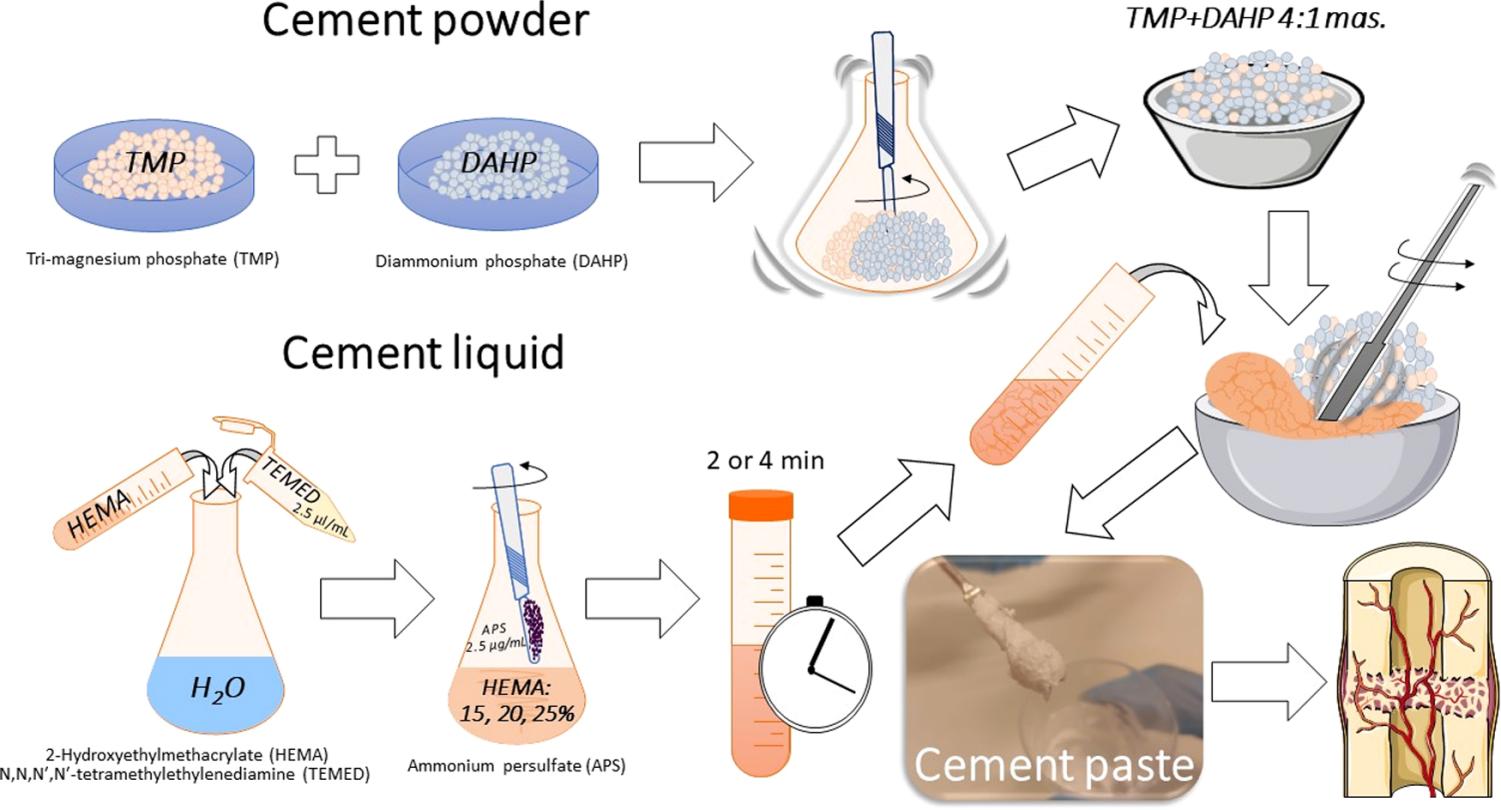
Schematic illustration of the preparation of MPC+HEMA bone cements.

Trimagnesium phosphate (TMP, Mg_3_(PO_4_)_2_) powder was obtained by sintering a mixture of MgHPO_4_·3H_2_O (Alfa Aesar) and Mg(OH)_2_ (VWR Prolabo) in 2:1 molar ratio at 1100 °C for 5h. The obtained sintered cake was manually crushed, ground dry in a planetary ball mill (PM400, Retsch GmbH, Germany), and sieved <355 μm. Then, it was sintered again and milled for 3 h to obtain the appropriate powder size of about 11.16 ± 5.67 μm. A control XRD study was performed during the process, and the particle size distribution was determined using a laser particle size analysis (L300, Horiba, Japan). 4 g of the resulting TMP powder was finally mixed with 1 g finely ground (20 s coffee grinder) diammonium hydrogen phosphate (DAHP, (NH_4_)_2_HPO_4_, ACS, Merck) and thoroughly mixed in a plastic bottle. The cement liquids were aqueous solutions of 2-hydroxyethyl methacrylate (HEMA, >99% with <50 ppm monomethyl ether hydroquinone as inhibitor, Merck) in the initial HEMA concentrations in the range 10–50% (H %), including 2.5 μL/mL *N*,*N*,*N*′,*N*′-tetramethylethylenediamine (TEMED, >99%, Sigma-Aldrich). 2.5 mg/mL ammonium persulfate (APS, >98%, Sigma-Aldrich) was added to the solutions to start the hydrogel polymerization reaction. The cement powder-to-liquid ratio was 2.5 g/mL, and different polymer premix times: 2 and 4 min (p2/p4) were applied. The cement preparation and composition, including the used raw materials (sintering regime, Mg/P ratio, and P/L ratio), was selected as optimal based on our previous studies,^39,40^ while the two different premix times were chosen by preliminary tests. The tested concentrations of HEMA were selected based on hardening time and compressive strength testing as the most favorable. The final six selected research groups of cement compositions are as follows: MPC + H15%_p2, MPC + H20%_p2, MPC + H25%_p2, MPC + H15%_p4, MPC + H20%_p4 and MPC + H25%_p4, and a sample photo of the obtained specimens is also included in Figure S1. The cement specimens were prepared by premixing HEMA solutions with an APS activator for a certain time and then adding them to cement powder in a plastic bowl and manually stirring until a homogeneous paste was obtained. Next, the paste was transferred into silicone rubber molds (in three dimensions: cubic: 6 × 6 × 12 mm, beam: 3 × 4 × 40 mm, and disk: 2 × 15 mm) and stored for 24 h at 37 °C and >90% humidity (water bath). As a reference, the cement powder was mixed with water and treated in a manner identical with that of the tested cements. The procedure for obtaining the proposed bone cements is shown in Figure 1.

### 2.2 Characterization

#### 2.2.1 Setting Time

The initial setting time of cement paste (*n* = 3) was measured qualitatively with a metallic dissecting needle (diameter 1.13 mm) and stopwatch starting at the moment of the two cement components combination. This time was considered as the length of time to the moment that specimens were fully solidified, and the indentation mark was not visible on the surface.

#### 2.2.2 Microstructure Analysis

The surface microstructure of obtained cement was examined by high-resolution scanning electron microscopy (SEM) DSM 940 (Zeiss, Germany) after being dried at 40 °C for at least 24 h. Before examination, all specimens were stuck on special holders via conductive stickers and then sputtered with a thin (4 nm) platinum layer for electron reflection.

#### 2.2.3 Phase and Chemical Composition

The cement specimens, after hardening, were crushed and ground in a mortar and then analyzed by a D8 X-ray diffractometer (XRD) in DaVinci design (Bruker, Germany). Data were collected from 2θ = 20–40° with a step size of 0.02° and a scan rate of 1.5 s/step using Cu–Kα radiation with a 40 kV voltage and a 40 mA current. Joint Committee on Powder Diffraction Standards (JCPDS) references were considered for XRD pattern evaluation, mainly reference patterns for farringtonite (PDF Ref 33-0876), struvite (PDF Ref 15-0762), and newberyite (PDF Ref 35-0780). The cement pastes during the setting reaction as well as dried cements were analyzed by Fourier transform infrared spectrometer (FTIR) Nicolet is10 (Thermo Fisher Scientific) in the range of 4000 to 650 cm^–1^ with 16 scans and a resolution of 4 cm^–1^. The spectra were acquired in absorbance mode, normalized, and smoothed.

#### 2.2.4 Porosity

The initial and final porosity Φ (%) of the cements (*n* = 3) were calculated by the following equation^41^
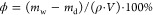
where *m*_d_ is the dry mass and *m*_w_ is the wet mass (g) after immersion in PBS (when a constant weight is achieved), ρ is the density of PBS (g/cm^3^), and *V* is the volume of the specimen (cm^3^).

#### 2.2.5 Surface Wettability

The surface wettability was determined on dry cement specimens by water contact angle measurements with an optical tensiometer (Attention Theta Life, Biolin Scientific, Finland) based on the falling drop method (volume ∼1 μL; *n* = 5).

### 2.3 Mechanical Properties

The static compressive and 3-point flexural tests (*n* = 5) were performed using a Universal Mechanical Testing Machine Z440 (Zwick, Germany) with a 10 kN load cell with a crosshead speed of 1 or 5 mm/min, respectively. An example photo taken during the study is shown in Figure S4. The compressive (σ_c_) and bending (σ_b_) strengths, as well as compressive (*E*_c_) and bending modulus (*E*_b_), were calculated by a standard method using integrated software. As there is no ISO standard for testing mineral bone cements, the mechanical testing regime was adapted whenever possible from the ISO5833:2022 standard for polymeric bone cements based on acrylic resin.^42^ Selected stress–strain curves for tested bone cements are shown in Figure S5.

### 2.4 Degradation Behavior

The dried and hardened cements (*n* = 3) were washed in 1 mL of phosphate-buffered saline (PBS) per specimen for 3 h (with a change of solution every hour) to remove possible salt residues in material pores. Then, the specimens were dried at 37 °C overnight and weighed (initial mass was determined). Finally, cements were immersed in 2.5 mL of PBS solution (Merck, Germany) and stored for 18 days at 37 °C with a PBS change every third day. After the immersion, specimens were removed from the solution, dried overnight, and weighed again (final mass was determined). The relative mass loss was calculated by the following equation^43^
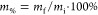
where *m*_%_ is the mass change (%), *m*_f_ is the final mass, and *m*_i_ is the initial mass (g). The analytical balance accuracy of the laboratory scale was 1.0 mg.

### 2.5 Microhardness after Degradation

The hardness of cements after 30 days of PBS exposure was determined by microindentation technique with NanoTestTM Vantage equipment (Micro Materials, U.K.) already applied for cements.^44^ The experiments were performed by using a three-sided diamond and a pyramidal indenter (Berkovich indenter). The following parameters were set up on the basis of experimental selection: 2000 mN of the maximum load, 20 s of the loading time, 15 s of the holding time, and 5 s of the holding time under maximum force. The microhardness (*n* = 10) was calculated by the Olivier–Pharr method^45^ using integrated software.

### 2.6 Cytocompatibility

Cell activity of obtained cements was evaluated using a human osteoblast cell line (hFOB 1.9; ATTC CRL-11372) cultured in F12/Dulbecco’s modified Eagle’s medium supplemented with 0.3 mg/mL Geneticin sulfate (G-418, Thermo Fisher Scientific) and 10% fetal bovine serum (Biowest, France) at 34 °C and 5% CO_2_. Before testing, all specimens (*n* = 4) were sterilized with 75% ethanol (1h) followed by exposure to UV light (2 × 30 min) and then immersed in 1 mL per specimen in the above-mentioned medium for 24 h. Afterward, the medium was discharged. The cells were seeded at a density of 40 × 10^3^ cells/mL on the surface of materials in 1 mL of fresh culture medium—direct test. Parallelly, the extracts of cements were done by immersing specimens in 1 mL of the culture medium—conditioned test. Then, this conditioned medium was used in the experiment. The cells were seeded at a 24-well plate at a density of 40 × 10^3^ cells/well, and the preliminary culture was 24 h. Then, the medium was changed to the conditioned one. The cell viability and lactate dehydrogenase (LDH) release were analyzed after 3 days of culture. During the time of the experiment, half of the culture medium was changed to a fresh one every day to equilibrate the ion level. The activity of LDH, as an indicator of cell death, was determined by direct measurement of NADH oxidation. The activity of the enzyme was calculated as nmol of produced NAD because of an absorbance coefficient for NADH = 6.22 mol/cm at 340 nm (Ultrospect 3000pro spectrophotometer; Amersham-Pharmacia-Biotech, Cambridge, U.K.). The results were normalized with a negative control incubated on neat cement MPC and a positive one (100% death) with 0.2% v/v Triton X-100. For cell viability, the culture medium was exchanged with a fresh medium, including MTT (thiazolyl blue tetrazolium bromide; Merck, Germany), and incubated for 4 h. The development of the colored product metabolized by living cells was assessed colorimetrically using a microplate reader (Victor, PerkinElmer) at 595 nm toward reference 690 nm. The results were normalized with a control incubated on neat cement MPC (100%).

### 2.7 Statistics

Statistical analysis of the data was performed using commercial software (SigmaPlot 14.0, Systat Software, San Jose, CA). The Shapiro–Wilk test was used to assess the normal distribution of the data. All of the results were calculated as means ± standard deviations (SD) and statistically analyzed using one-way analysis of variance (one-way ANOVA). Multiple comparisons versus the control group between means were performed using the Bonferroni *t* test, with the statistical significance set at *p* < 0.05.

## 3 Results

### 3.1 Setting Time

The setting time of bone cement is one of the critical application parameters and is directly related to the hydration reaction speed and the hardening of the material itself. Pure MPC had a setting time in the range of 20.63–24.63 min, and the addition of HEMA significantly reduced this time. The obtained results are shown in Table 1. As the HEMA content increased, the setting time was shortened, depending on the content, by approximately 3–5 min. However, the different tested premix times had no significant effect on this parameter.

**Table 1:** Setting Time of the Tested Bone Cements (*n* = 3; Data Are Expressed as the Mean ± SD)^b^

	**setting time [min]**
MPC	22.55 ± 1.92
MPC+H15%_p2	19.65 ± 1.62
MPC+H20%_p2	19.02 ± 1.05^a^
MPC+H25%_p2	17.72 ± 1.42^a^
MPC+H15%_p4	18.92 ± 1.27^a^
MPC+H20%_p4	18.50 ± 1.02^a^
MPC+H25%_p4	17.32 ± 1.32^a^

a Statistically significant difference as compared to control–MPC (*p* < 0.05).

b Statistically significant difference between premix times (*p* < 0.05).

### 3.2 Microstructure Analysis

**Figure 2:**
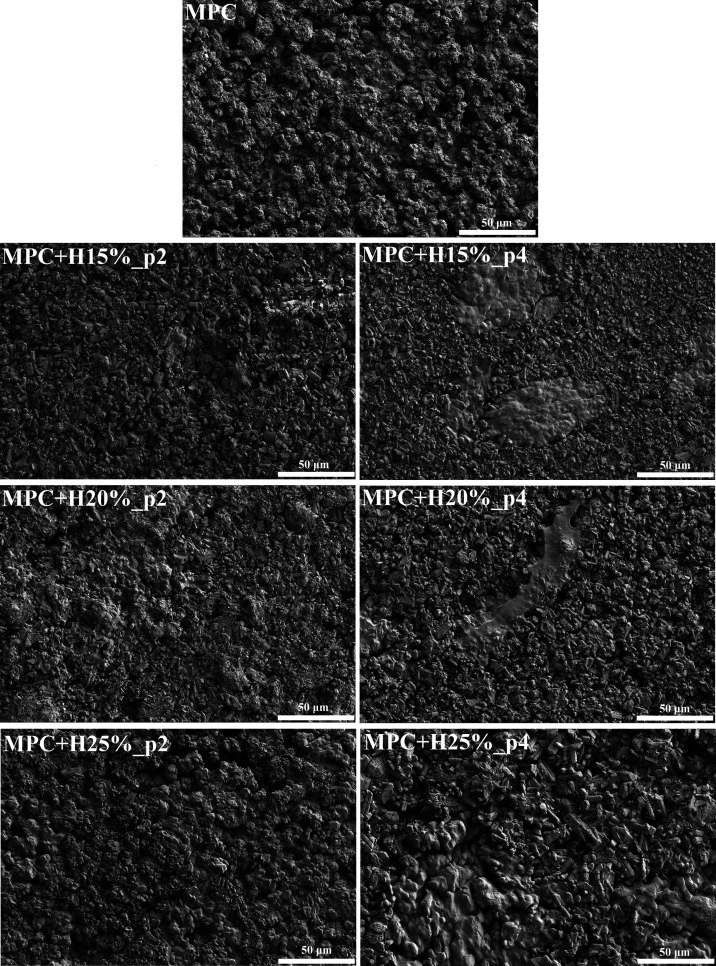
SEM images of the tested bone cements at 500× magnification after curing for 48 h (the pictures are representative of five specimens).

The morphology of the hardened cement is shown in Figure 2, and the pure cement consists mainly of magnesium phosphate rodlike crystals with a size of about ∼2–5 μm linked to each other with a gel-like surface in a specific cement matrix. It may be observed that some of the crystals are cracked, which may be related to the drying process. As the content of HEMA increases, its more significant share in the morphology of cements was observed. This polymer component is bound to the cement crystals and forms gel-like clusters in the structure. Longer premix times significantly influenced the creation of larger HEMA agglomerates. Further, it can be observed that the pHEMA phases are not homogeneous in the specimens, especially for longer premix times.

### 3.3 Phase and Chemical Composition

**Figure 3:**
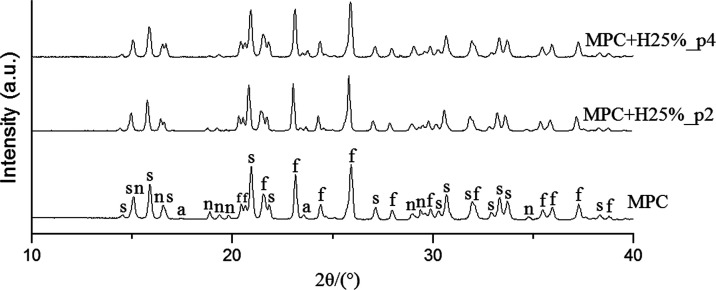
XRD patterns of the tested bone cements after curing for 24 h under 37 °C, 100% humidity. Characteristic reflexes are marked as “s” (struvite), “n” (newberyite), “f” (farringtonite), and “a” (ammonium hydrogen phosphate).

The XRD spectra of investigated cements are shown in Figure 3 (and also in Figure S2), and the corresponding XRD patterns showed that both pure cement and those modified with HEMA consisted of three well-crystallized phases, which are referred to as struvite (NH_4_MgPO_4_ × 6 H_2_O, PDF 15-0762), newberyite (MgHPO_4_ × 3 H_2_O, PDF 19-0762), and farringtonite (Mg_3_(PO_4_)_2_, PDF 33-0876). Moreover, traces of unreacted ammonium hydrogen phosphate (PDF 20-0091 or 22-0051) were found in all cements. We found no differences in peak shifts and their intensity. Therefore, it may be concluded that the addition of HEMA did not negatively affect the hydraulic reaction of MPC.

**Figure 4:**
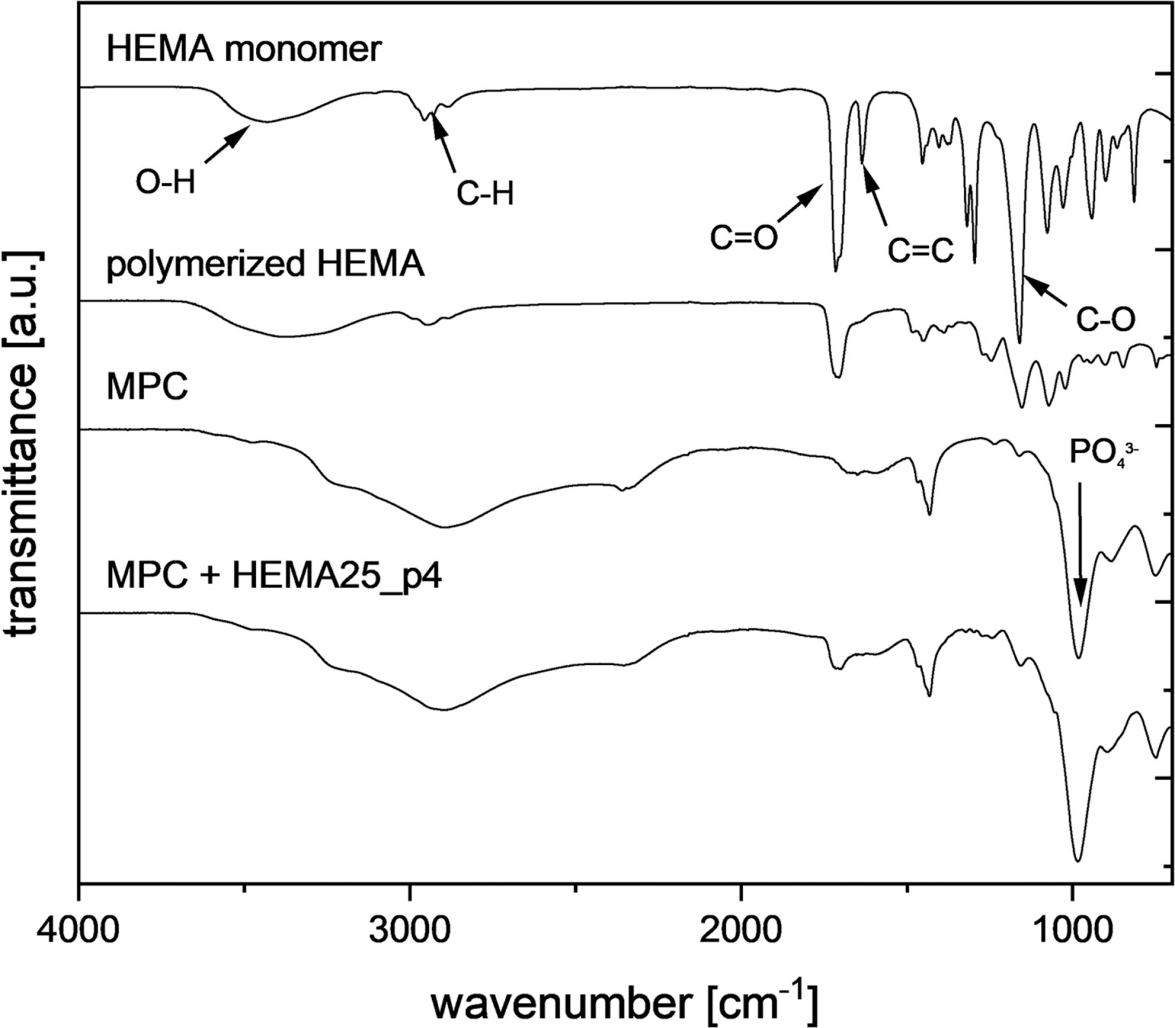
FTIR spectra of the tested bone cements after hydrogel polymerization.

As shown in Figure 4, the FTIR spectra of HEMA exhibited the following bands typical for methacrylate hydrogels: −OH (∼3422 cm^–1^), C–H (2954–2888 cm^–1^), C=O (∼1712 cm^–1^), C=C (∼1647 cm^–1^), C–H (∼1456 cm^–1^), and C–O (∼1160 and ∼1022 cm^–1^), whereas the polymerization reaction resulted in a strong decrease of the intensity of the C=C band.^33^ Pure MPC exhibited mainly a broad peak contributed to PO_4_^3–^ (∼1010 and ∼950 cm^–1^). In composite cements, FTIR spectra adequate to pHEMA and MPC were found. Hence, it is possible to confirm that the cross-linking of the HEMA hydrogel has also taken place in the cement matrix.

### 3.4 Porosity

**Figure 5:**
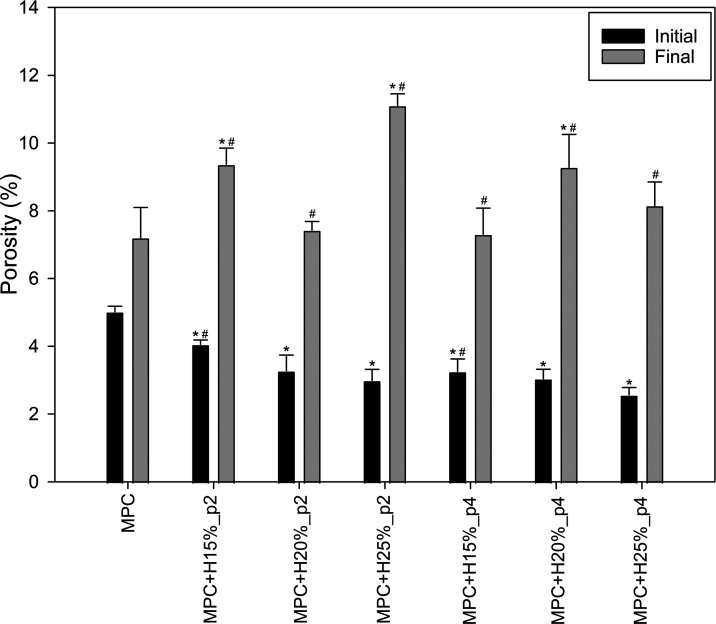
Initial (after hardening) and final (after PBS incubation) porosity of the tested bone cements (*n* = 3; data are expressed as the mean ± SD; * statistically significant difference as compared to control–MPC (*p* < 0.05), # statistically significant difference between premix times (*p* < 0.05)).

As shown in Figure 5, the initial porosity of modified cements decreased with increasing HEMA content; however, a different effect was observed for the final porosity. Due to significant discrepancies in final porosity, no clear correlation between HEMA content and premix time was observed, but generally, this additive had a positive effect on the cement’s porosity.

### 3.5 Degradation Behavior

Table 2 shows the degradation behavior of cements determined by weight loss. Both premix time and HEMA content influenced the degradation of the samples. The most significant mass loss was observed for specimens with shorter premix times.

**Table 2:** Mass Loss (after Incubation in the PBS Solution) of the Tested Bone Cements (n = 3; Data Are Expressed as the Mean ± SD)

	mass loss [%]
MPC	0.346 ± 0.253
MPC+H15%_p2	3.038 ± 0.442^a^^b^
MPC+H20%_p2	3.864 ± 0.423^a^^b^
MPC+H25%_p2	2.987 ± 1.041^a^^b^
MPC+H15%_p4	0.297 ± 0.197^b^
MPC+H20%_p4	0.519 ± 0.383^b^
MPC+H25%_p4	1.179 ± 0.370^a^^b^

a Statistically significant difference as compared to control–MPC (*p* < 0.05).

b Statistically significant difference between premix times (*p* < 0.05).

### 3.6 Surface Wettability

**Figure 6:**
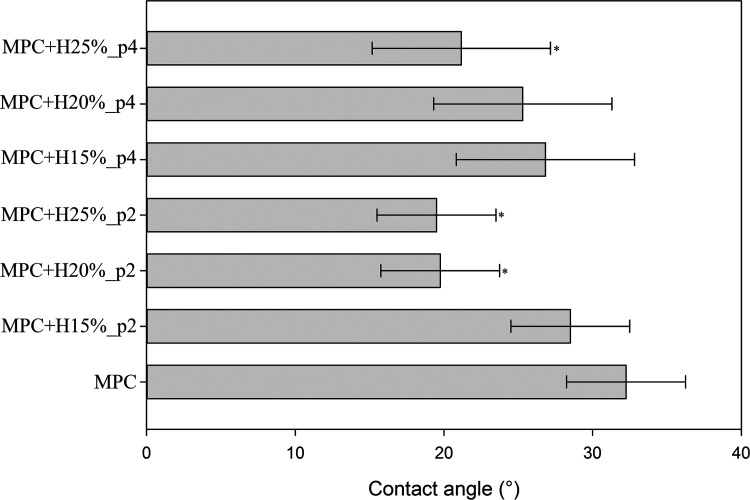
Surface wettability of the tested bone cements determined by the measurements of the contact angle of distilled water (*n* = 5; data are expressed as the mean ± SD; * statistically significant difference as compared to control–MPC (*p* < 0.05), # statistically significant difference between premix times (*p* < 0.05)).

All tested cements have good wettability (contact angles of ∼20–30°), as shown in Figure 6. An increase in HEMA content slightly improved the wettability of cements, whereas premix time did not affect this property. Example images from the analysis are shown in Figure S3.

### 3.7 Mechanical Properties

**Figure 7:**
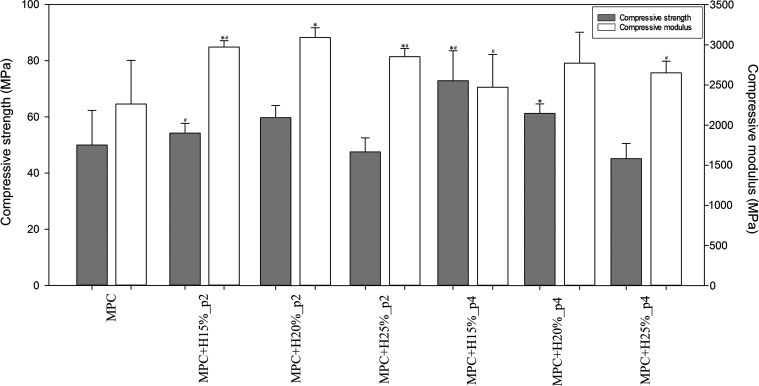
Compression strength of the tested bone cements (*n* = 10; data are expressed as the mean ± SD; * statistically significant difference as compared to control–MPC (*p* < 0.05), # statistically significant difference between premix times (*p* < 0.05)).

**Figure 8:**
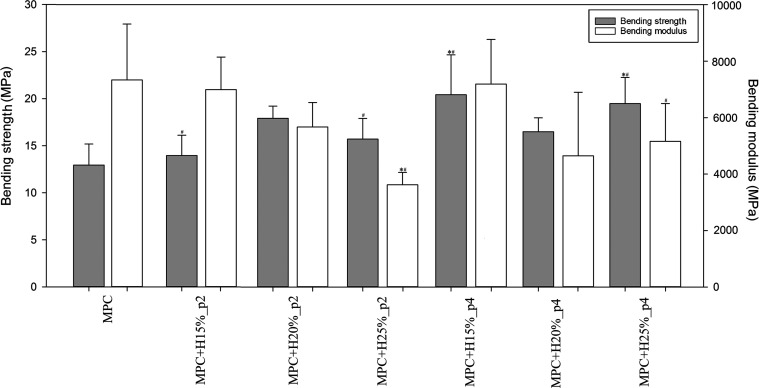
Bending strength of the tested bone cements (*n* = 10; data are expressed as the mean ± SD; * statistically significant difference as compared to control–MPC (*p* < 0.05), # statistically significant difference between premix times (*p* < 0.05)).

As shown in Figures 7 and 8, the addition of HEMA had a significant effect on the mechanical properties of cements. The pure magnesium cement had a compressive strength of ∼50 MPa and bending strength of ∼13 MPa,^46^ while for HEMA-modified cements, an increase in these values was obtained in most cases (CS: ∼ 45–73 MPa, BS: ∼13–20 MPa; except MPC+H25%). Particular improvement has been observed for cements with a longer premix time and lower HEMA contents, especially MPC+H15%_p4. Moreover, the compressive modulus for modified cements has increased in any case, especially significant for shorter premix time, while the bending modulus did not change or significantly decrease, and higher HEMA content exacerbated this effect, especially MPC+H25%_p2.

### 3.8 Microhardness after Degradation

The hardness test by the microindentation method focused mainly on the assessment of susceptibility to local plastic deformation and was applied to evaluate changes in mechanical properties of tested modification after a month of degradation (Table 3). It was observed that a more significant deterioration of microhardness occurred for specimens with a longer premix time, especially MPC+H25%_p4 and MPC+H15%_p4. In the case of a shorter premix time, only the specimens with the highest HEMA content (MPC+H25%_p2) had a significant reduction in this mechanical property.

**Table 3:** Microindentation Properties after 30 Days of PBS Exposure (*n* = 10; Data Are Expressed as the Mean ± SD)

hardness after PBS exposure		
	[GPa]	% of control
MPC	0.104 ± 0.019	100% ± 18%
MPC+H15%_p2	0.096 ± 0.017^b^	92% ± 15%^b^
MPC+H20%_p2	0.097 ± 0.015	93% ± 14%
MPC+H25%_p2	0.073 ± 0.022^a^	70% ± 21%^a^
MPC+H15%_p4	0.068 ± 0.017^a^^b^	65% ± 16%^a^^b^
MPC+H20%_p4	0.089 ± 0.012	86% ± 11%
MPC+H25%_p4	0.055 ± 0.029^a^	53% ± 28%^a^

a Statistically significant difference as compared to control–MPC (*p* < 0.05).

b Statistically significant difference between premix times (*p* < 0.05).

### 3.9 Cytocompatibility

**Figure 9:**
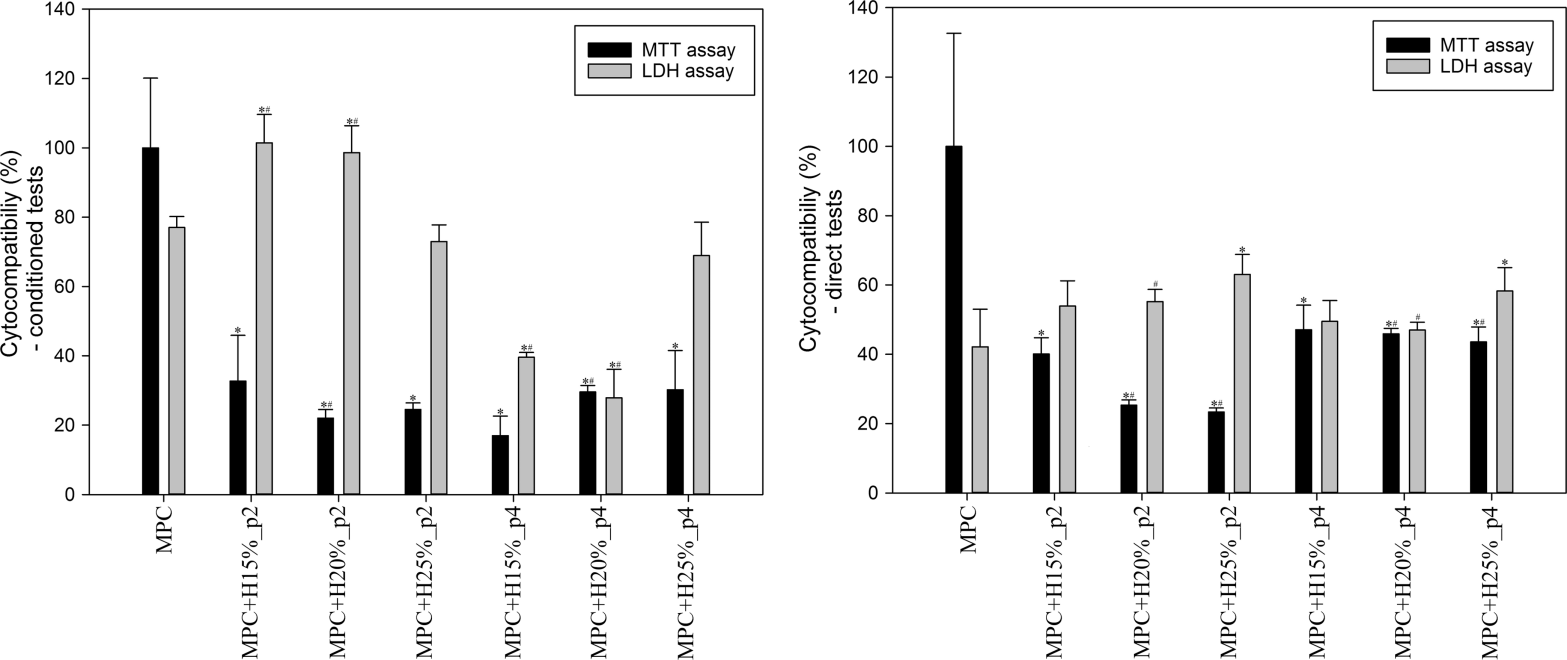
Cytocompatibility of the tested bone cements (*n* = 4; data are expressed as the mean ± SD; * statistically significant difference as compared to control–MPC (*p* < 0.05), # statistically significant difference between premix times (*p* < 0.05)).

In order to assess the cytocompatibility of modified cements, two types of studies were carried out: a conditioned test and a direct test on the material, and the results are shown in Figure 9. All cements containing HEMA significantly decreased cell viability, and the effect was cytotoxic. The cell death was confirmed by LDH assay—the enzyme release was significantly increased in the cells treated with MPC+HEMA both in direct exposure and in conditioned tests. The most cytotoxic effects (MTT: ∼ 22–25%) for both tests were obtained for shorter premix time and higher HEMA contents (H20 and H25%, p2), while cements with lower HEMA content and shorter premix time (H15%, p2) showed less toxicity to hFOB cells (MTT: ∼36% in both types of testing). The extended premix time and the increased HEMA content (p4, H20 and H25%) also contribute to the reduction of the cytotoxic effect (MTT: ∼30% conditioned tests or ∼45% direct tests). Meanwhile, LDH release from the nonliving cells in the direct test was elevated by 10, 12, and 21% for shorter premix time and the addition of HEMA 15, 20, and 25%, respectively, compared to MPC. The extension of premix time did not elevate the cellular mortality compared to MPC, except from the MPC+H25%; however, this cytotoxic effect was weaker than exerted by shorter premix time. Additionally, cytocompatibility tests of the poly-HEMA hydrogels themselves were carried out in accordance with the applied above methodology, and those results confirmed the significant toxicity for those cement components (MTT: ∼2–3% conditioned tests or ∼5–10% direct tests; data not included).

## 4 Discussion

Novel dual-setting composite bone cements were effectively obtained for all applied HEMA contents and two tested premix times, which was confirmed by microstructure analysis and evaluation of chemical and phase composition. While the previous fabrication of dual-setting α-TCP/HEMA cements was relatively simple by mixing only one slowly setting cement component with the aqueous HEMA cement liquid,^34^ transferring this concept to MPC+HEMA cements was much more challenging. Here, the cement usually consists of two components, farringtonite and a highly concentrated diammonium phosphate solution. Since it was impossible to mix HEMA monomer with the ammonium phosphate solution, the salt had to be added in dry form to the cement powder. Second, the setting of MPC is relatively fast and consumes a large amount of water from the cement liquid to form the highly hydrated mineral struvite. Since this competes with the hydrogel formation during HEMA polymerization and leads to a phase separation in the cement paste after mixing, the polymerization reaction had to be initiated prior to the addition of the cement component. This was achieved by premixing HEMA solution with the initiator systems APS and TEMED for either 2 or 4 min (based on preliminary tests) before adding the cement powder, which then resulted in workable cement pastes with appropriate setting times for clinical application in range ∼16–22 min (Table 1);^47^ the hardened cements consisted of well-crystallized phases of struvite, newberyite, and farringtonite (Figure 3) as well as a polymerized hydroxyethyl methacrylate (Figure 4); their microstructure was characteristic for ceramic cements^48^ with visible magnesium phosphate crystals and separated areas of gel-like clusters of poly(HEMA) (Figure 2). The cements showed low porosity, below ∼4% after production and below ∼11% after 1 month of degradation (Figure 5), with an adequate wettability (Figure 6)^49^ and depending on the HEMA content as well as premix time, different mechanical properties, i.e., compression and bending strength and microhardness (Figures 7 and 8, Table 3), and various degradation rate (Table 2).

### Influence of HEMA Content

Three HEMA contents were selected from the range of 10–50% optimal for MPC cement modification and thoroughly characterized in this work: 15, 20, and 25%. In preliminary studies, it was observed that such values of polymer show an appropriate hardening time of dual-setting cement and the most significant differences in mechanical properties. The increase in HEMA content resulted in the shortening of cement setting time (Table 1). In most works on modification of MPC, it was found that the addition of polymer extended its setting time, which was related to the prolongation of the dissolution step of cement raw materials.^36^ In our research, we observed the opposite phenomenon, which may be related to the use of a reactive polymeric system, which increases viscosity during polymerization and therefore contributes to cement hardening. The microstructure of dual-setting cements was also different depending on the content of HEMA (Figure 2). With an increasing content, more polymer agglomerates in the cement matrix were formed. Similar results were obtained in the work of Christel et al.^33^ and Hurle et al.^34^ for α-TCP+HEMA cements. In our study, no effect of HEMA content on both setting reactions was confirmed (Figures 3 and 4), and similar observations regarding the creation of composite cements and no negative impact on its setting reactions were observed, for example, in the work of Liao et al. based on MPC and chitosan.^37^ Increasing the HEMA content contributed to a decrease in the initial porosity of the cement, which may be related to the filling of free spaces between the MgP crystals and pores by the polymer component. Adequate conclusions were proposed by Gong et al. in research on MPC with incorporated oxygen-carboxymethyl chitosan.^38^ The relationship between the HEMA content and the rate of degradation was also observed, but it does not seem to be linear and is strongly dependent on the premix time (Table 2). This factor and the very heterogeneous microstructure contributed strongly to the different final porosity after 1 month of PBS exposure (Figure 5); however, no apparent effect of HEMA content was found. The surface’s wettability was also dependent on the HEMA content, and its increase resulted in a decrease in the contact angle (Figure 6). The polymer had a contact angle value of approximately 50–60°,^33^ and MPC had ∼32°, while the combination of these two components contributed to lowering the contact angle and improving wettability. Different HEMA contents also affected the mechanical properties of cement. All specimens containing HEMA showed an improvement in bending strength and lowering of bending modulus, incredibly significant for higher HEMA contents (Figure 8). However, in the case of compressive strength (Figure 7), only lower contents (15 and 20%) had a positive effect, while the 25% HEMA content already caused a compressive strength decrease. The mechanical properties of materials are also dependent on their internal porosity. Here, the initial porosity of the tested groups differed by ∼1.0–2.5%, which may also slightly result in a weakening of their mechanical strength. However, no clear trend between these properties was observed, and a greater influence of HEMA addition on this property is suspected. Compressive modulus increased in all cases, most preferably for the medium HEMA content. The most significant reduction in microhardness after degradation was found for specimens with the highest HEMA content. Further, it should be remembered that in the case of this research, the surface of specimens must be perpendicular to the indenter tip; otherwise, results may be inaccurate.^50^ Here, due to the diverse surface of MPC cement, it is possible that results may be slightly over or underestimated. The effect of the polymer on the composite material may vary and depends on the type of polymer as well as its distribution in the matrix. It is assumed, however, that poly-HEMA improves the elastic properties of the ceramics and reduces the number of cracks in the matrix by preventing crack propagation.^51,52^ Such improvement of mechanical properties with lower contents of the polymer additive was previously noted in the literature.^36^ MPC, depending on the preparation, has a compressive strength of 10–50 MPa, while human cortical bone has about 90–190 MPa.^53^ Therefore, the improvement of mechanical properties is crucial in the aspect of using those cements as somewhere load-bearing implants. In previous research works on MPC modification by polymer addition, it was possible to improve this strength close to ∼50–60 MPa values,^35,36,52^ and here, our developed dual-setting cement has a compressive strength even more improved, about ∼73 MPa (MPC + H15%_p4).

### Influence of HEMA Premix Time

The additional premix time of HEMA polymerization was used to eliminate the phase separation problem in the cement paste. At the stage of preliminary research, times in the range of 1–6 min were tested, and finally, it was decided to choose two premix times for more detailed research: 2 and 4 min. It was found that the premix time shortened the setting time of the cement (Table 1). This may be due to the formation of a polymer gel that absorbed water and reduced its availability in the cement reaction, which shortened the hydration time. The relationship between cements reaction time and liquid-to-powder ratio has been previously confirmed in studies by Ma et al.^54^ Further, extending the premix time significantly affected the formation of poly-HEMA agglomerates in the cement structure (Figure 2). It can also be observed that the pHEMA phases are not uniformly distributed in the structure. This might be due to the partial demixing of pHEMA from the aqueous solution during polymerization, which is likely more pronounced at longer premix times. No differences in terms of chemical and phase bonds were observed (Figures 3 and 4). Longer premix times slightly decreased cement porosity, except MPC+H20% (Figure 5). It was also found that extending this time significantly affected the degradation of cement and reduced its mass loss during incubation in PBS solution (Table 2). Similar results were obtained by Zarybnicka et al. in research on MPC with cross-linked poly(vinyl alcohol).^35^ No significant differences in the wettability of cements with different premix times were observed (Figure 6). It has been observed that the premix time has a significant positive effect on the mechanical properties of cements and allows for significantly improved compressive and bending strength of the MPC + H15% cement (Figures 7 and 8). In the case of compressive modulus, there was a slight decrease for the MPC+H15% and H25%, while in the case of bending modulus, there was an increase for MPC+25%. The mechanical properties after degradation tested by microhardness confirmed the significant effect of the premix time on the weakening of strength (Table 3). These differences may result from different arrangements as well as the number and size of polymer agglomerates in the ceramic matrix. Such observations have been previously reported for biocomposites.^55^

### Selection of the Most Favorable Dual-Setting Cement

Cement MPC+H15%_p4 was characterized to have the most favorable properties, such as improved compressive strength by ∼45.8% (∼72.9 MPa) and bending strength by ∼57.9% (20.4 MPa); most minor cytotoxic effect on osteoblastic cells in direct tests (∼47%); shortened setting time (∼18:55); and more appropriate gel-like handling characteristic and better formability. In this work, the key objective was to improve the mechanical properties of MPC, and this cement showed the most significant mechanical strength. For bone substitutes applied in osteoporotic bone, this parameter is critical to avoid early phase collapse.^56^ The setting time of this cement is suitable for preparing the paste during surgery and its application in the defect.^47^ Improvement of the formability of gel-like paste may enable, in the future, after additional optimization, the application of this cement in the form of injection, which is essential because minimally invasive operations are more preferred.^57,58^

### Cytocompatibility and Future Research Perspective

In general, it is assumed that modern biomaterials should actively support the adhesion, proliferation, and differentiation of surrounding bone cells such as osteoblasts, osteoclasts, and osteocytes.^41^ Research on MPC confirms its high effectiveness in treating bone defects through active bone regeneration.^59^ Here, our MPC cements generally showed a negative effect on cell viability, as confirmed by the LDH release ratio (compared to TCP); however, this may be related to the high ion release of MPC during degradation. This phenomenon was previously observed by Li et al.,^60^ who confirmed that incubation of magnesium phosphate in the culture medium affects ion concentration, especially Mg and Ca, and that disturbance of the culture conditions may cause cell death. Furthermore, the addition of HEMA also contributed to the significant cytotoxicity of cements (compared to MPC) under *in vitro* conditions. There is no direct correlation between cement cytocompatibility and HEMA content, but a longer premix time significantly improved the hFOB cell viability (Figure 9), except for MPC+H15%_p2 in conditioned tests. This may be related to the more efficient polymerization reaction obtained during the additional premix time and the greater stability of the hydrogel, which releases less amount of cytotoxic compounds. Such observations are consistent with the research of Mironi-Harpaz et al. on photopolymerized hydrogels.^61^ Further, porosity also has a significant impact on cell viability;^62^ on the one hand, it affects their adhesion, but it also could contribute to the release of substances from the matrix. Our additional biological studies confirmed that the toxic effect is related to the applied polymerization using TEMED+APS. Hence, increased porosity, also due to the faster biodegradation, could contribute to more significant cytotoxicity, but such a trend between these properties was not found in our study. The toxic effects of poly(HEMA) were previously observed, for example, by Morisbak et al.^63^ Also, Desai et al. tested and confirmed that those polymerization agents are a significant source of toxicity for cells.^64^ In this study, the degree of HEMA polymerization was not evaluated, which is a limitation regarding the hypotheses put forward above. However, in our further work, we assume optimization of our cement technology or even the use of a different polymerization system, such as, i.e., benzoyl peroxide/ascorbic acid.^65^ Further, Kim et al. developed a novel poly(HEMA-Am) hydrogel polymerized with the TEMED+APS method, which was fully cytocompatible.^66^ Therefore, such a solution may also be applied to our developed dual-setting cements. In general, the problem with *in vitro* testing of MPC cements is a known phenomenon, while their high biocompatibility has already been confirmed in *in vivo* research.^20^ It remains clear, however, that further biocompatibility studies of proposed dual-setting cements are crucial for their potential clinical use, and the need for more thorough *in vivo* research is a limitation of this work.

## 5 Conclusions

In the present study, we successfully developed a novel dual-setting bone cement based on a combination of magnesium phosphate cement with poly(2-hydroxyethyl methacrylate). The addition of polymer significantly influenced various properties of the cement but did not negatively affect its phase structure, and magnesium phosphate was obtained. The cement consisted of well-crystallized phases and polymerized HEMA, had a favorable setting time (close to ∼16–21 min), low porosity (up to ∼11%), and adequate wettability (∼20–30°), and its microstructure was highly diverse. Further, our study demonstrates that both production parameters, the concentration of HEMA (15–25%) and its premix time (2–4 min), allow us to obtain a cement with a variable microstructure and different characteristics, especially mechanical strengths. Bone cement based on MPC with 15% of HEMA and 4 min of premix time seems to be a favorable candidate for potential clinical application as its compressive and bending strength was improved (∼72.9 and 20.4 MPa, respectively), was hardened in ∼19 min, and was characterized by more appropriate gel-like handling characteristic and better formability. However, there was a problem regarding the biocompatibility of the developed cements, and in the future, we will work on optimization of the polymerization process to eliminate the negative impact of the reaction initiators.

## Data Availability

Data will be made available on request.

## Supporting Information Available

Additional experimental details for dual-setting bone cement based on MPC+pHEMA, including photographs of the obtained specimens, supplementary experimental methods (of wettability and mechanical tests), as well as accurate results of XRD patterns for all tested groups and compressive curves (PDF)

## Abbreviations

APS: ammonium persulfate
ANOVA: analysis of variance
BC: bone cement
BS: bending strength
CaP: calcium phosphate
CS: compression strength
DAHP: diammonium hydrogen phosphate
FTIR: Fourier transform infrared spectrometer
hFOB: human osteoblast cell line
HEMA: 2-hydroxyethyl methacrylate
JCPDS: Joint Committee on Powder Diffraction Standards
LDH: lactate dehydrogenase
MPC: magnesium phosphate cement
MTT: thiazolyl blue tetrazolium bromide
PBS: phosphate-buffered saline
pHEMA: poly(2-hydroxyethyl methacrylate)
PMMA: poly(methyl methacrylate)
SEM: scanning electron microscopy
SD: standard deviations
TEMED: *N*,*N*,*N*′,*N*′-tetramethylethylenediamine
TMP: trimagnesium phosphate
XRD: X-ray diffractometer
