# Supplementary material for: Dual-Setting Bone Cement Based On Magnesium Phosphate Modified with Glycol Methacrylate Designed for Biomedical Applications
Source: ACS Appl Mater Interfaces. 2023 Nov 21;15(48):55533–44. doi: 10.1021/acsami.3c14491 (PMC10711722; doi:10.1021/acsami.3c14491)
Supplement: Supplementary file 1 — am3c14491_si_001.pdf [file am3c14491_si_001.pdf]

Supporting Information

Dual-setting bone cement based on magnesium phosphate modified with glycol methacrylate designed for biomedical applications

Marcin Wekwejt[*a], Maryia Khamenka[b], Anna Ronowska[c] and Uwe Gbureck[d]

[a]*Biomaterials Technology Department, Faculty of Mechanical Engineering and Ship Technology, Gdańsk University of Technology, G. Narutowicza 11/12 Street, 80-233, Gdańsk, Poland*
[b]*Scientific Club 'Materials in Medicine', Advanced Materials Centre, Gdańsk University of Technology,*
G. Narutowicza 11/12 Street, 80-233, Gdańsk, Poland
[c]*Chair of Clinical Biochemistry, Department of Laboratory Medicine, Medical University of Gdańsk, Gdańsk, M. Skłodowskiej-Curie 3a Street, 80-210, Gdańsk Poland*
[d]*Department for Functional Materials in Medicine and Dentistry, University of Würzburg, Pleicherwall 2 Street, D-97070 Würzburg, Germany*
[*] marcin.wekwejt@pg.edu.pl

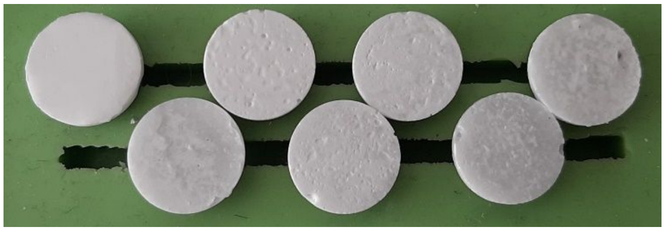

Fig. S1. Sample photo of tested bone cements from different groups after curing 24h under 37°C, 100% humidity.

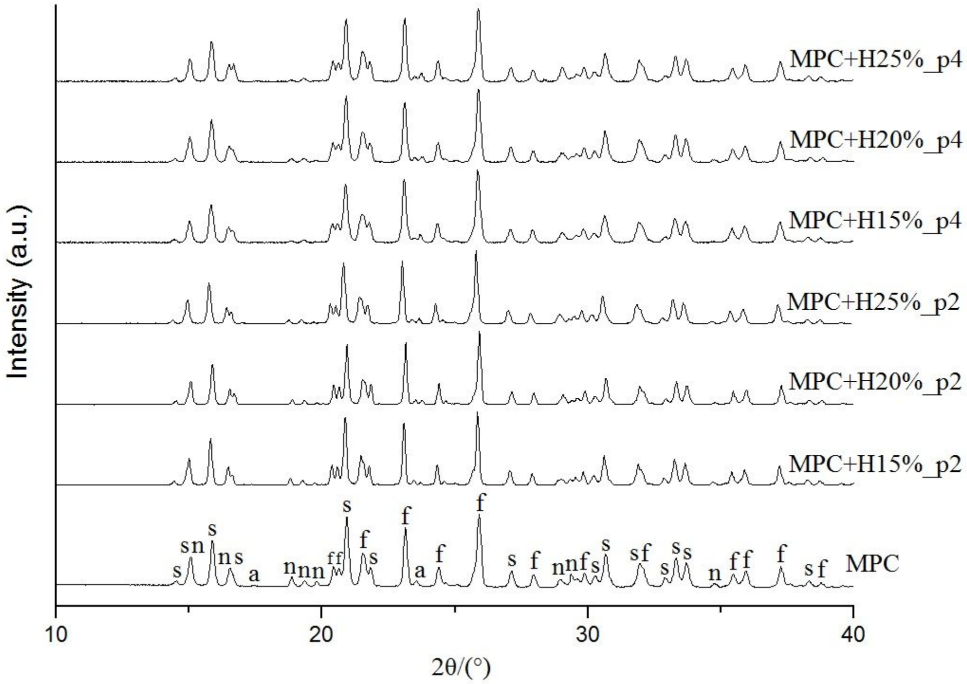

Fig. S2. XRD patterns of all tested bone cements after curing 24h under 37°C, 100% humidity. Characteristic reflexes are marked as: 's' (struvite), 'n' (newberyite), 'f' (farringtonite) and 'a' (ammonium hydrogen phosphate)

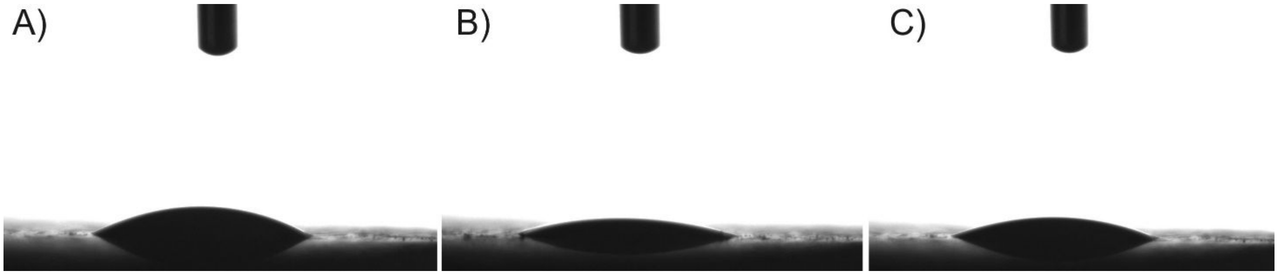

Fig. S3. Example images of water contact angle measurements for the tested bone cements: A) MPC, B)MPC+H25%_p2, C) MPC+H25%_p4

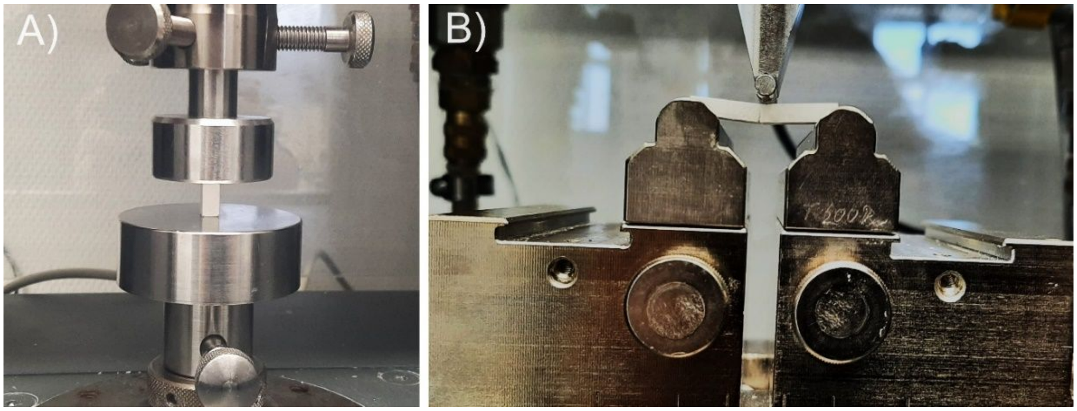

Fig. S4. Example photo taken during the mechanical properties research: A) static compression test and B) static bending test

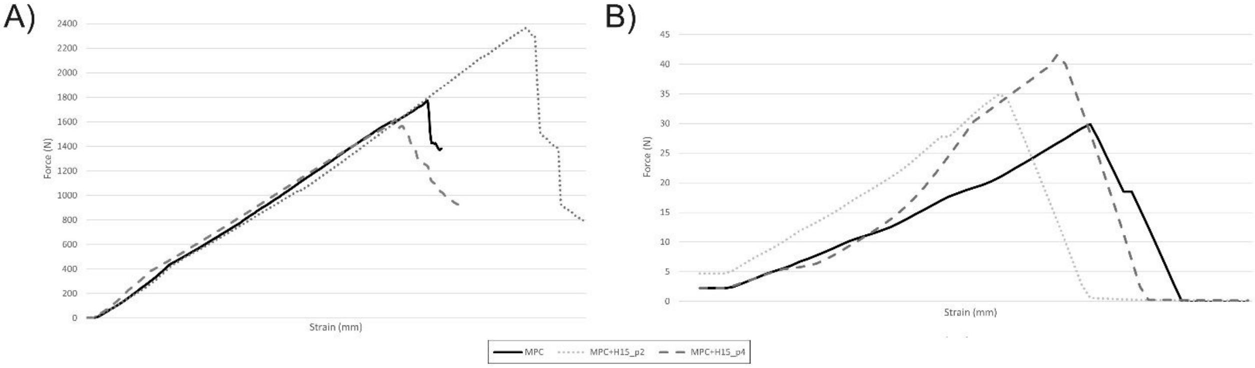

Fig. S5. Selected mechanical curves for the tested bone cements with the most favorable properties (MPC+H15): A) compression test and B) bending test
